# Long Non-Coding RNAs: Allies and Enemies of HIV Infection

**DOI:** 10.32607/actanaturae.27881

**Published:** 2026

**Authors:** T. F. Kikhai, A. V. Mashkovskaia, T. S. Oretskaya, Y. Y. Agapkina, M. B. Gottikh

**Affiliations:** Lomonosov Moscow State University, Faculty of Chemistry, Moscow, 119991 Russia; Belozersky Institute of Physico-Chemical Biology, Lomonosov Moscow State University, Moscow, 119991 Russia; Lomonosov Moscow State University, Faculty of Bioengineering and Bioinformatics, Moscow, 119991 Russia

**Keywords:** long non-coding RNA, human immunodeficiency virus, viral replication, latency

## Abstract

significant impact on the pathogenesis of viral infections by acting as both positive and negative regulators of viral gene expression. This review summarizes the available data on lncRNAs associated with human immunodeficiency virus type 1 (HIV-1). LncRNAs can regulate both the active replication cycle and the latent phase of the HIV-1 infection, during which the integrated proviral DNA remains transcriptionally silent. Moreover, lncRNAs may serve as diagnostic markers and are potential therapeutic targets. A deeper understanding of the intricate interactions between lncRNAs and HIV-1 is essential for developing innovative treatments for the HIV infection and the associated acquired immunodeficiency syndrome (AIDS).

## INTRODUCTION

Human immunodeficiency virus type 1 (HIV-1) remains a major global health challenge. Despite remarkable advances in antiretroviral therapy (ART), which have made it possible to manage the HIV-1 infection and prevent the development of acquired immunodeficiency syndrome (AIDS), complete eradication of the virus from infected individuals remains an unattainable goal. A major barrier to viral eradication is the formation of a stable latent reservoir: a pool of infected cells harboring integrated proviral DNA in a transcriptionally silent state, rendering it inaccessible to both the immune system and antiretroviral drugs. A key breakthrough in recent years has been the finding that HIV does not passively “hide” in stochastically quiescent cells, but it actively programs them to enter a quiescent state [1]. This process involves the activation of the cellular transcription factors KLF2 (Krüppel-like factor 2) [2] and p53, which suppress the main cellular “engine”; namely, MYC (a proto-oncogene and transcription factor) [1]. Without MYC, the cell enters a temporary state of quiescence, and the virus in it turns silent, forming a stable latent reservoir [1].

Various strategies are being developed to overcome that latency. The so-called “shock-and-kill” strategy is the most studied approach so far; its aimed is to reactivate the provirus using latency-reversing agents (LRAs), followed by elimination of infected cells either by the immune system or through virus-induced cytolysis [3]. Unfortunately, none of the studied LRAs has yet yielded the desired outcome due to the heterogeneity of latent reservoirs, toxicity, or insufficient efficacy [3]. An alternative approach, the “block-andlock” strategy, seeks to sustainably suppress viral transcription, thereby “sealing” the virus in a latent state [4]. Immune-based therapeutic strategies are being developed as well; they include the use of monoclonal antibodies and genetically engineered T cells (CAR-T cells) for a targeted elimination of infected cells [5].

The effectiveness of these approaches directly depends on a comprehensive understanding of the complex molecular mechanisms governing HIV-1 transcription and latency. A key role in this regulation is assigned to long non-coding RNAs (lncRNAs): a class of transcripts longer than 200 nucleotides (nt) that do not encode proteins. Instead, lncRNAs act as key epigenetic, transcriptional, and post-transcriptional regulators and participate in chromatin organization, splicing control, mRNA stabilization, and the modulation of intracellular signaling pathways, including the innate immune response.

The current review summarizes available data on the role of various lncRNAs in the HIV-1 life cycle. The impact of lncRNAs on viral replication, as well as on the establishment and maintenance of latency, is discussed. A better understanding of the multiple functions of lncRNAs opens up new possibilities for the development of therapeutic agents aimed at overcoming latency and achieving a complete cure for the HIV-1 infection.

## THE HIV-1 REPLICATION CYCLE AND LATENCY

Immune system cells expressing CD4 receptors on their surface, primarily CD4^+^ T cells, as well as monocytes, macrophages, and some other cells, are the main targets of HIV-1 in the human body [6, 7, 8]. Upon infection, the viral capsid, which contains viral genomic RNA and viral enzymes, is released into the infected cell cytoplasm. Complementary DNA (cDNA) is synthesized by the viral reverse transcriptase within the capsid. In the cell nucleus, cDNA is integrated into the cellular genome by viral integrase. Next, RNA transcription, splicing, and translation occur; all these processes are effectuated by the host cell machinery. The resulting viral proteins and new genomic RNAs are assembled into new virions and released from the cell. Drugs comprising modern ART act at different stages of the viral life cycle that involve viral proteins: viral entry into the cell, reverse transcription, integration, processing of viral polyproteins, and assembly of the viral capsid [9, 10, 11, 12, 13]. This therapy prevents the formation of new virions and reduces the viral load in infected individuals, which significantly improves the quality of life and life expectancy of HIV-infected patients.

However, after the integration of viral cDNA into the cellular genome, some cells enter a quiescent state, during which active transcription from the integrated viral DNA and production of new virions do not occur [14]. Epigenetic mechanisms, primarily deacetylation and trimethylation of the histones associated with viral DNA, play a crucial role in the establishment of viral latency [15]. It is for this reason that drugs affecting the epigenetic landscape (e.g., histone acetyltransferase/deacetylase inhibitors) can either induce or reverse latency. Latently infected cells form HIV-1 reservoirs [16]. ART drugs have no effect on the latent virus. This limitation of ART, which acts only on a few stages involving viral proteins in cells with active viral replication, explains the need for a detailed exploration of the cellular components involved in the HIV infection, including lncRNAs.

Although epigenetic modifications (histone deacetylation and trimethylation at the long terminal repeat (LTR) regions) play a key role in latency formation and maintenance, there are also other complementary mechanisms. For instance, limited availability of the transcription factors NF-κB (nuclear factor kappa B) and NFAT (nuclear factor of activated T cells) is observed in resting cells. These transcription factors are located in the cytoplasm in an inactive form and do not enter the nucleus, which prevents the initiation of transcription from the LTR promoter [17, 18]. Furthermore, integration into transcriptionally active genes may result in transcriptional interference, when elongating RNA polymerase II traverses the provirus and suppresses its expression, especially in a certain integration orientation; however, the results may vary depending on the cellular model [18, 19, 20]. Low levels of P-TEFb and the absence of Tat in quiescent cells prevent RNA polymerase II from overcoming the elongation pause, thereby suppressing productive proviral transcription [21].

Oxidative stress may be involved in the regulation of HIV-1 latency. Although prooxidant signals can stimulate provirus transcription through the activation of NF-κB and AP-1, the virus paradoxically hijacks the host’s antioxidant pathways (including the thioredoxin and glutathione systems) to maintain persistence and protect infected cells from apoptosis [22]. Thus, redox homeostasis acts as a bidirectional regulator of HIV-1 latency, while the balance between oxidation and reduction determines the infection’s fate. All of these mechanisms can act simultaneously, creating a heterogeneous pool of latently infected cells.

Considering that the transcriptional stage is crucial for both productive HIV-1 replication and latency maintenance/reversal, we will first discuss those HIV-1-associated lncRNAs that modulate transcription from the HIV-1 promoter. This is the most numerous group of lncRNAs involved in the HIV infection.

## HIV-1 PROMOTER

**Fig. 1:**
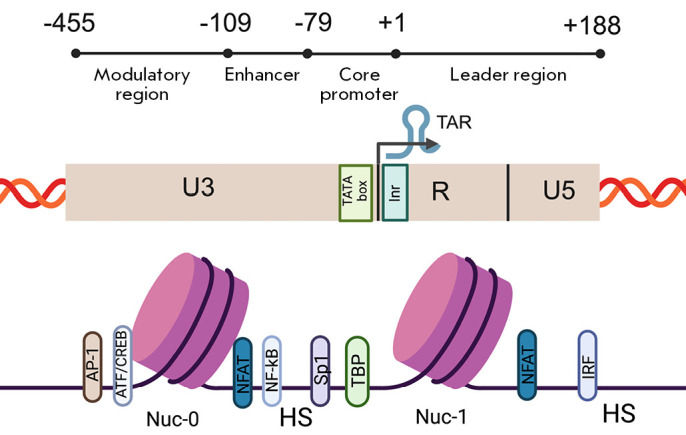
Schematic representation of the HIV-1 5’-LTR structure highlighting key regulatory regions: the modulatory region, enhancer, core promoter, and leader region. Main binding sites for cellular transcription factors (NF-κB, Sp1, TBP, NFAT, AP-1, CREB, and IRF), which are critical in initiating viral genome transcription and reactivation from latency, are shown

The viral DNA integrated into the cellular genome contains so-called long terminal repeats (LTRs) at its ends. Three main regions can be distinguished in the LTR structure: U3, R, and U5 (Fig. 1). Transcription initiation takes place at the boundary between regions U3 and R in the 5’-LTR. U3 contains the viral promoter recognized by RNA polymerase II and a number of regulatory elements. Four functional regions can be distinguished in the 5’-LTR: the modulatory region (nucleotides -455…-109), enhancer (-109…-79), core promoter (-78…-1), and the leader region (+1 (the transcription start site) ...+188) [23]. These regions contain binding sites for cellular transcription factors. NF-κB, Sp1, and the TATA-boxbinding protein (TBP) are considered the most important factors for transcriptional activation (Fig. 1). Factors AP-1, IRF, ATF, CREB, and NFAT are also important, since mutations in their binding sites drastically reduce the efficiency of viral transcription and replication [24].

The 5’-LTR enhancer region contains binding sites for NF-κB, which is an extremely important factor for the activation of HIV-1 transcription and virus reactivation from latency [25, 26]. As a result of transcription initiation by NF-κB, short ~60–80-nt transcripts carrying a stable hairpin structure called TAR (trans-activation response element) at the 5’ end are synthesized. After TAR RNA formation, transcription pauses, since RNA polymerase II is arrested by binding to NELF (negative elongation factor) and DSIF (5,6-dichloro-1-β-D-ribofuranosylbenzimidazole sensitivity-inducing factor) [23]. Initiation of transcription elongation requires phosphorylation of all of the above factors, as well as the C-terminal domain of RNA polymerase II. This is achieved through the action of the transcription elongation factor P-TEFb (positive transcription elongation factor b), which consists of cyclin-dependent kinase 9 (Cdk9) and cyclin T1 (CycT1). In cells containing the latent virus, P-TEFb is predominantly maintained in an inactive state due to binding to the small nuclear ribonucleoprotein complex 7SK (7SK snRNP, small nuclear ribonucleoprotein), which inhibits the kinase activity of P-TEFb and prevents transcription elongation [23].

Transcription elongation is primarily regulated by the viral protein Tat (trans-activator of transcription). Tat binds to TAR RNA and recruits P-TEFb to the latter, thereby inducing phosphorylation of RNA polymerase II and the factors NELF and DSIF [27]. Therefore, a decrease in the Tat protein level or its complete absence in cells during viral latency negatively affects transcription efficiency, thereby contributing to the maintenance of viral latency [28].

**Long non-coding RNAs activating transcription**

MALAT1. MALAT1 (metastasis-associated lung adenocarcinoma transcript 1) is an evolutionarily conserved lncRNA of ~8,000 nt that is transcribed from the 11q13.1 locus [29]. Despite its oncology-related name, MALAT1 is not a tumor marker; it is expressed in normal human tissues, including lungs, pancreas, prostate, ovaries, and brain, where it regulates alternative splicing and gene expression [30].

Recent studies have revealed an important role for MALAT1 in HIV-1 pathogenesis. MALAT1 levels are significantly elevated in peripheral blood mononuclear cells (PBMCs) from HIV-1-infected individuals not receiving ART compared to patients receiving ART with undetectable viral loads [31]. This indicates a direct association between viral replication and MALAT1 expression. Experiments in various cell models have also shown that low MALAT1 levels are associated with a dramatic decrease in HIV-1 replication efficiency, since this lncRNA stimulates transcription from the LTR promoter [31].

An analysis of the effect of MALAT1 on the latent virus revealed that, in latently infected U1 monocytic cells, viral reactivation using the histone deacetylase inhibitor SAHA, which promotes chromatin decondensation and activates transcription, results in a significant increase in the expression of both viral RNA and MALAT1 [31].

Another mechanism by which MALAT1 promotes HIV-1 reactivation from latency has been described [32]. An important mechanism involved in maintaining latency is trimethylation of histone H3 at lysine 27 (H3K27me3) within nucleosomes Nuc1 and Nuc2 at the LTR promoter [33]. The methyltransferase EZH2 (enhancer of zeste homolog 2), a catalytic subunit of the Polycomb repressive complex 2 (PRC2), is responsible for the establishment of this epigenetic mark [34]. In CD4^+^ T cells, MALAT1 directly interacts with EZH2 and thereby displaces the latter from the HIV-1 promoter, preventing H3K27me3 deposition [32]. In the absence of H3K27me3, the promoter becomes more accessible to transcription factors [25] and RNA polymerase II, which further enhances transcription and promotes viral reactivation from latency [32].

**Fig. 2:**
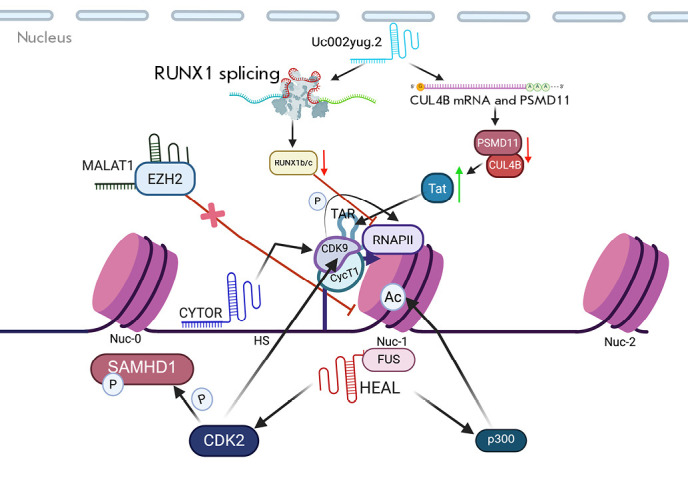
Visualization of the mechanisms by which lncRNAs (MALAT1, HEAL, CYTOR, and uc002yug.2) promote HIV-1 replication and latency reversal. The model demonstrates several key processes: recruitment of transcriptional activators (p300 and P-TEFb) to the LTR promoter; activation of the P-TEFb subunit CDK9 and inactivation of SAMHD1 through phosphorylation; displacement of repressive complexes (PRC2 and EZH2); increase of Tat protein levels via suppression of its proteasomal degradation, and a shift in the epigenetic landscape toward active transcription

The importance of MALAT1 for latent virus activation is confirmed by a study that utilized CRISPR screening to confirm the transcription factor ETS1 as a regulator of HIV-1 latency that represses MALAT1 gene expression [35]. ETS1 knockout in latently infected cells increases MALAT1 levels, thus promoting viral reactivation, and also increases the levels of transcriptionally active H3 histone marks (H3K9ac, H3K27ac, and H3K4me3) at the LTR promoter. This confirms the existence of a complex regulatory network in which ETS1 acts as an “ally” of latency by reducing cellular MALAT1 levels [35] (Fig. 2).

MALAT1 levels change not only during the HIV infection but also in other physiological and pathological conditions. Cellular MALAT1 levels change at strictly defined stages of the cell cycle and during differentiation; they increase under hypoxia and other stress conditions; this process is mediated by the transcriptional regulators HIF-2α, CREB, and Sp1 [36]. MALAT1 levels are elevated in many malignant neoplasms (lung, breast, liver, and prostate cancers) and correlate with an unfavorable prognosis and metastasis [36]. Thus, changes in MALAT1 expression during the HIV-1 infection may reflect not only virus-specific effects but also more general cellular stress responses, which can be exploited by the virus to maintain its replication and latency

Another mechanism by which MALAT1 modulates HIV-1 replication is associated with the ability of this lncRNA to modulate the cellular immune response, which will be discussed in the relevant section.

***HEAL.*** HEAL (HIV-enhanced ncRNA) is a 441-nt lncRNA that plays a crucial role in the HIV-1 life cycle. It is transcribed from a gene located at the 1p35.3 locus [37]. HEAL is found only in humans, chimpanzees, and rhesus macaques, which suggests its recent emergence and a possible involvement in HIV-1 species specificity [38].

HEAL expression is significantly upregulated in various HIV-1 target cells, including monocyte-derived macrophages (MDMs), microglia, and T cells, as well as in PBMCs from HIV-infected individuals compared to uninfected donors, indicating its potential clinical significance as a biomarker [38]. In cells with reduced HEAL levels, HIV-1 replication is significantly suppressed, which confirms the role of HEAL as a key positive regulator of HIV-1 replication [38, 39].

The ability of HEAL to activate transcription of the latent virus has been studied in a model of latently infected cells incubated in the presence of the antiretroviral drug azidothymidine (AZT), followed by withdrawal of AZT treatment. AZT withdrawal in cells with normal HEAL levels resulted in pronounced viral reactivation, while transcription from the provirus in cells with either suppressed or undetectable HEAL expression did not resume even after 28 days. This result indicates that HEAL is required for viral reactivation from latency [38].

A clinical study conducted on a cohort of HIVinfected individuals who interrupted therapy showed that HEAL levels in patient PBMCs significantly increased upon viral reactivation and decreased upon therapy resumption [39].

A study investigating the mechanism underlying HEAL proviral activity showed that this lncRNA forms a complex with the cellular protein FUS (fused in sarcoma) [38]. This complex acts through two main mechanisms. Firstly, the complex binds to the DNase I hypersensitive site (DHS), which is virtually free of histones, and to the Nuc-1 nucleosome in the 5’-LTR, thus recruiting the histone acetyltransferase p300. This leads to H3K27ac deposition, an epigenetic modification associated with active transcription. This is accompanied by enhanced recruitment of the positive transcription elongation factor P-TEFb to the promoter. P-TEFb phosphorylates DSIF, NELF, and the C-terminal domain of RNA polymerase II [40], thus ensuring efficient elongation of viral transcription [38].

Secondly, the HEAL–FUS complex binds to the promoter of the cyclin-dependent kinase 2 (CDK2) gene and activates its expression. Elevated CDK2 protein levels promote HIV-1 replication by phosphorylating and inactivating SAMHD1 (SAM and HD domain-containing protein 1), which dephosphorylates the deoxynucleoside triphosphates required for DNA synthesis during reverse transcription, as well as by activating the CDK9 catalytic subunit of P-TEFb, thereby further enhancing viral transcription [38].

Thus, the formation of the HEAL–FUS complex is a positive factor in terms of both increasing viral infectivity through stimulation of reverse transcription (since SAMHD1 inactivation increases the pool of deoxynucleoside triphosphates) and enhancing viral transcription and reactivation from latency through transcription activation (Fig. 2).

***CYTOR.*** CYTOR (cytoskeleton regulator RNA), also known as LINC00152, is a lncRNA encoded by a gene at the 2p11.2 locus [41]. Stimulation of latently infected CD4^+^ T cells with the protein kinase C activator phorbol 12-myristate 13-acetate (PMA) and ionomycin, which together mimic T-cell receptor activation and induce downstream signaling pathways, results in nuclear translocation of NF-κB and NFAT. This is a standard in vitro model for the reactivation of latent HIV-1. The use of PMA and ionomycin to reactivate latent HIV is widely documented [42]. Stimulation with these substances elevates CYTOR expression in cells with activated viral transcription (Fig. 2).

A study of the mechanism of CYTOR action showed that this lncRNA activates transcription through two mechanisms. The first mechanism involves CYTOR binding directly to the HIV-1 LTR promoter and recruiting P-TEFb, which further promotes transition to transcription elongation [43]. Having bound to the promoter, CYTOR also modulates its epigenetic landscape by increasing the enrichment of the active transcription marks H3K27ac and H3K4me3; however, the mechanism of this process is not discussed in the aforementioned study

In the second mechanism, CYTOR regulates the expression of the genes responsible for the organization of the actin cytoskeleton. For instance, a decrease in CYTOR expression is accompanied by reduced polymerization of cortical actin in response to T-cell receptor engagement [43]. Considering that inhibition of actin polymerization suppresses HIV-1 replication and prevents the activation of resting T cells, the ability of CYTOR to regulate actin polymerization makes this lncRNA a promising target for the reactivation of latent HIV-1.

***Uc002yug.2.*** Uc002yug.2 is a 2,564-nt-long lncRNA that is transcribed from a gene located at 21q22.12 [37]. The lncRNA uc002yug.2 is an important regulator of both replication and reactivation of latent HIV-1 [44]. The levels of this lncRNA in HIV-infected cells positively correlate with viral replication and infectivity. It has been demonstrated that uc002yug.2 regulates HIV-1 transcription, which enables it to promote viral reactivation from latency.

The lncRNA uc002yug.2 utilizes two main mechanisms of transcription activation. Firstly, it promotes alternative splicing of the cellular transcription factor RUNX1 mRNA, resulting in decreased levels of its isoforms RUNX1b and RUNX1c, thereby relieving repression of transcription from the LTR promoter [44]. Secondly, uc002yug.2 independently upregulates the viral transcriptional transactivator Tat, which is required for productive transcription of HIV-1 genes. Interestingly, this RNA does not affect Tat mRNA levels, while an increase in Tat protein levels is achieved by reducing the expression of mRNAs encoding components of the proteasome degradation system: proteins CUL4B (a component of an E3 ubiquitin ligase) and PSMD11 (a regulatory subunit of the 26S proteasome). In other words, a uc002yug.2-mediated increase in the intracellular Tat protein level is achieved through suppression of its proteasomal degradation (Fig. 2).

Weakening of transcriptional repression, together with enhanced transcriptional activation, promotes efficient viral reactivation from latency. The most important evidence of uc002yug.2 activity is that its elevated levels in resting CD4^+^ T cells from three HIV-infected individuals receiving ART induce a pronounced reactivation of the latent virus, comparable to that of the activators PMA and SAHA (suberoylanilide hydroxamic acid, a histone deacetylase inhibitor) [44]. The combination of PMA (protein kinase C activator) and SAHA synergistically enhances HIV-1 reactivation by inducing NF-κB signaling and chromatin decondensation [45]. It should also be noted that the expression level of this lncRNA in HIV-infected patients not receiving ART was found to be significantly higher than that in HIV-infected individuals receiving therapy for more than three years and maintaining undetectable plasma viral loads [44].

**Long non-coding RNAs repressing transcription**

***NRON.*** NRON (non-coding repressor of NFAT) is a ~2,700-nt-long lncRNA encoded at the 9q33.3 locus [46]. Its main cellular function is to regulate the transcription factor NFAT, which is expressed in the majority of immune cells [46]. NFAT is known to activate transcription from the HIV-1 LTR promoter [24, 47]. Therefore, it is reasonable to suggest that NRON also participates in the regulation of viral gene transcription. The effect of NRON on HIV-1 replication has been studied in lymphoid cells. During the early stages of the HIV-1 infection, a significant downregulation of intracellular NRON expression, followed by its subsequent upregulation, is observed in cells [48]. Viral proteins Nef and Vpu act during the early and late stages of infection, respectively; Nef suppresses NRON transcription, whereas Vpu promotes it. Changes in intracellular NRON levels affect the efficiency of transcription from the viral promoter through the interaction between NRON and NFAT. NRON directly binds NFAT in the cytoplasm and prevents its translocation to the nucleus. Hence, during the early stage of infection, the NRON level is reduced, leading to NFAT accumulation in the nucleus and transcription activation, whereas increased NRON levels result in NFAT retention in the cytoplasm and decreased transcriptional efficiency [48].

**Fig. 3:**
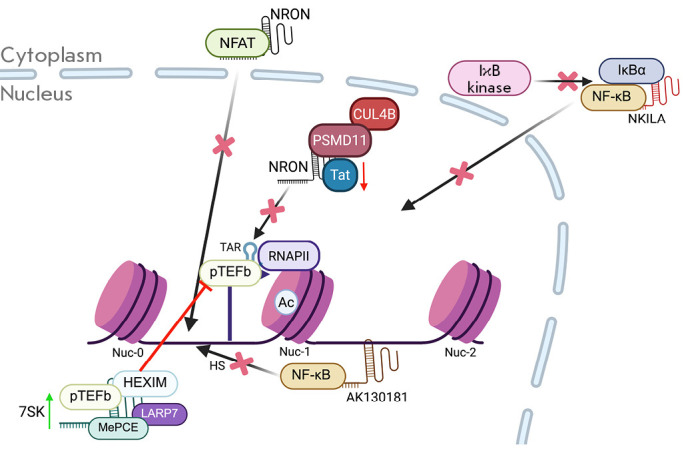
Illustration of the mechanisms of HIV-1 transcription suppression by antiviral lncRNAs (NRON, 7SK, NKILA, and AK130181). The model demonstrates how these molecules maintain viral latency by sequestering key transcription factors (NF-κB and NFAT) in the cytoplasm, inhibiting the P-TEFb elongation factor in 7SK snRNP, and directing the Tat viral protein toward proteasomal degradation

In latently infected cells, NRON is highly expressed, suggesting that it can bind NFAT in the cytoplasm, thereby reducing transcriptional efficiency [49]. Another mechanism by which this lncRNA regulates HIV-1 transcription during latency has also been established: NRON binds the viral transcriptional activator Tat and subsequently forms a complex with CUL4B and PSMD11, which, as mentioned above, are components of the ubiquitin–proteasome degradation pathway [50]. This mechanism leads to decreased Tat levels due to its degradation and contributes to the maintenance of HIV-1 latency [50] (Fig. 3). Interestingly, NRON in this context acts antagonistically to uc002yug.2, which rescues Tat from the actions of CUL4B and PSMD11.

***7SK.*** Small nuclear RNA 7SK (snRNA 7SK) is transcribed by RNA polymerase III from a single functional gene located at the 6p12.2 locus. This highly conserved RNA is found in many vertebrate species; it is 331 nt long in humans [51, 52]. The primary function of 7SK is the negative regulation of the transcription factor P-TEFb. This snRNA serves as a scaffold for the assembly of the multisubunit nuclear ribonucleoprotein complex 7SK snRNP. In addition to snRNA 7SK, this complex comprises the proteins HEXIM1 (or HEXIM2), LARP7, and MePCE. Direct inhibition of the P-TEFb kinase activity is mediated by HEXIM1, which acquires its inhibitory function by interacting with 7SK [53, 54]. Binding to the 7SK/HEXIM1 complex inactivates P-TEFb, thereby inhibiting transcription elongation by RNA polymerase II [53, 54, 55].

The HIV-1 Tat protein utilizes a structural mimicry mechanism to compete with HEXIM1 for binding to 7SK and release P-TEFb from the inhibitory complex [56]. The snRNA 7SK consists of four major hairpin (stem-loop) structures (SL1, SL2, SL3, and SL4) connected by unstructured regions and a series of additional small hairpins. The first hairpin, SL1, binds proteins containing arginine-rich regions via the so-called arginine sandwich motif (ASM), in which nucleotides are arranged in a sandwich-like structure and form stacking interactions with the guanidinium moiety of arginine residues in the protein [56]. HEXIM1, as part of the 7SK snRNP complex, binds to this region of SL1. Unlike HEXIM1, Tat contains an additional arginine residue (R52), which enables it to displace HEXIM1 from 7SK snRNP and bind P-TEFb [56]. Tat further transfers P-TEFb to the viral RNA TAR, forming an active transactivation complex at the HIV-1 promoter, which dramatically enhances the transcriptional elongation of the viral genome [56].

Understanding the molecular mechanism underlying Tat-mediated transcriptional activation allows for the development of targeted agents that either interfere with this mechanism (the “block-and-lock” strategy) or activate it (the “shock-and-kill” strategy). For instance, short RNA oligonucleotides mimicking the 7SK functional domain have been designed to stabilize the inhibitory P-TEFb/HEXIM1 complex, prevent Tat binding to P-TEFb, and thereby suppress viral transcription [57]. In addition, high-affinity aptamers that selectively bind and inactivate P-TEFb, as well as nuclease-resistant small interfering RNAs (siRNAs) and antisense oligonucleotides aimed at 7SK degradation and virus reactivation, are being developed [57].

Another approach to the modulation of the 7SKdependent mechanism of transcriptional regulation is the development of highly specific inhibitors of the interaction between 7SK and the DKC1 protein. The concept is based on the fact that pseudouridinylation of U250 in 7SK is an important mechanism of posttranscriptional regulation of 7SK snRNP stability and function [58]. This modification is catalyzed by the pseudouridine synthase DKC1 [58]. Disrupted pseudouridylation destabilizes the 7SK snRNP complex, leading to the release of active P-TEFb and enhanced transcription from P-TEFb-dependent promoters, including the HIV-1 promoter (Fig. 3) [58].

Fine-tuning of the 7SK snRNP complex function and its maintenance are ensured at the stage of posttranslational modifications. The protein component HEXIM1 is phosphorylated at Ser158 by protein kinase C. The addition of a phosphate group prevents HEXIM1 from recruiting 7SK RNA and maintaining the elongation factor P-TEFb in an inactive state. In this regard, stimuli that activate protein kinase C prevent complete assembly of 7SK particles and increase P-TEFb-dependent gene expression [59]. In addition, HEXIM1 phosphorylation at Tyr271 and Tyr274 regulates P-TEFb release from the complex. Mutations Y271E and Y274E disrupt 7SK snRNP assembly and cause displacement of CDK9 (as a P-TEFb component) to the cytoplasm [60].

LARP7 also protects 7SK snRNA from degradation. The methylphosphate capping enzyme MePCE is tightly bound to 7SK. Upon interaction with LARP7, the enzyme loses its capping activity and acquires a new role: together with LARP7, it stabilizes 7SK and maintains the integrity of the 7SK snRNP complex [61]. Therefore, several mechanisms and signaling pathways that regulate this complex are often dysregulated in the HIV infection.

***AK130181.*** The level of lncRNA AK130181 (also known as LOC105747689) is significantly increased in HIV-1-infected resting CD4^+^ T cells compared to activated cells. This lncRNA was found to suppress transcription from the LTR promoter [62]. Smallinterfering RNA-mediated knockdown of AK130181 significantly reactivates viral transcription in latently infected Jurkat cells and primary CD4^+^ T cells. In contrast, AK130181 overexpression suppresses transcription from the LTR promoter. A study of the mechanism of action of this lncRNA showed that AK130181 directly interacts with the transcription factor NF-κB and, presumably, prevents its binding to the LTR promoter [62]. These findings suggest that AK130181 maintains HIV-1 latency by suppressing NF-κB activity (Fig. 3). It is important to note that AK130181 knockdown, in combination with the histone deacetylase inhibitor SAHA, synergistically enhances viral reactivation, suggesting that AK130181 may also contribute to the epigenetic regulation of HIV-1 latency [62].

NKILA. NKILA (NF-κB-interacting long non-coding RNA) is a 2,570-nt-long lncRNA that is transcribed from the 20q13 locus [63]. NKILA acts as an HIV-1 replication suppressor and also prevents viral reactivation from latency. Like AK130181, NKILA acts through NF-κB-dependent suppression of transcription [64]. In quiescent cells, NF-κB is retained in the cytoplasm through interaction with the inhibitory protein IκBα, which masks its nuclear localization signal. However, upon stimulation and reactivation from latency, IκB kinases IKKα and IKKβ phosphorylate IκBα, promoting its dissociation from the NF-κB complex. Once released from IκBα, active NF-κB is imported into the nucleus, where it interacts with its binding sites within the 5’-LTR and activates transcription initiation. NKILA can directly interact with the p65 subunit of the NF-κB complex and thereby bind to the NF-κB/IκBα complex in the cytoplasm. In the NKILA/NF-κB/IκBα complex, IκBα phosphorylation sites are inaccessible to the IκB kinase. Since IκBα can dissociate from the NF-κB complex only in its phosphorylated form [65], NF-κB cannot translocate from the cytoplasm to the nucleus and bind to the κB sites within the HIV-1 LTR promoter [26] (Fig. 3). This mechanism of NKILA action was confirmed by chromatin immunoprecipitation. Interestingly, this lncRNA also interferes with the interaction between the transcription factor NFAT and its target promoters; i.e., it exhibits an activity similar to that of the lncRNA NRON [48].

The virus itself counteracts the inhibitory effect of NKILA. NKILA expression is significantly reduced in CD4^+^ T cells during both an acute HIV infection and viral reactivation from latency [63]. This effect is primarily due to reduced H3K27ac enrichment at the NKILA promoter in the presence of HIV-1, which suppresses NKILA transcription. Thus, NKILA is an important component of the host’s innate immune response and its suppression by HIV-1 highlights the dynamic interplay between the virus and the host’s defense system.

**Functions of lncRNAs in the regulation of HIV-1 intracellular transport**

This section considers two lncRNAs. The first lncRNA, NEAT1, regulates the nuclear export of mRNA transcripts synthesized from the viral promoter. The second lncRNA, LINC02453, is responsible for the transport of the viral capsid from the cytoplasm to the nucleus.

NEAT1. NEAT1 (nuclear-enriched abundant transcript 1) is a ~4,000-nt lncRNA transcribed from the 11q13.1 locus [66]. NEAT1 plays a key role in the formation of paraspeckles: nuclear bodies located in the interchromatin space of the mammalian cell nucleus and involved in the post-transcriptional regulation of gene expression [66, 67]. Paraspeckles are responsible for the retention in the nucleus of RNA molecules containing double-stranded regions and subjected to multiple events of adenosine residue deamination. By regulating gene expression, paraspeckles participate in various cellular processes such as cell differentiation, response to stress, and viral infections [67]. For instance, HIV-1 exploits paraspeckles as a reservoir for retaining unspliced viral mRNAs [66, 68].

**Fig. 4:**
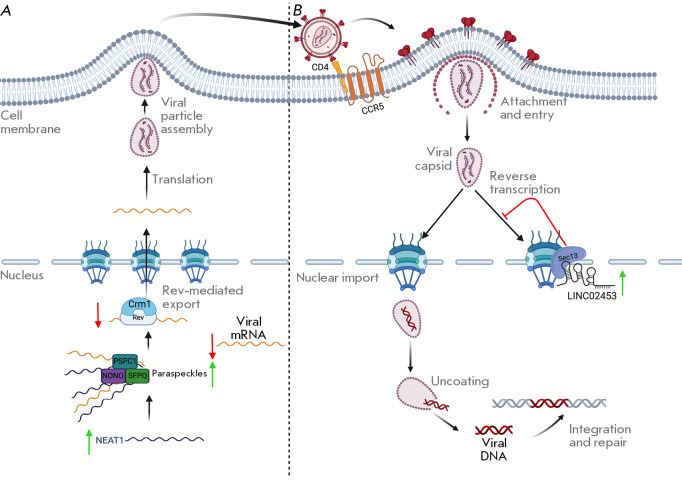
A schematic representation of the impact of lncRNAs on the intracellular transport of HIV-1 components. (A) NEAT1 forms paraspeckles and thus retains unspliced viral mRNAs in the nucleus, limiting their export. (B) LINC02453 lncRNA binds to the nuclear pore protein SEC13 and prevents viral capsid entry into the nucleus, thereby inhibiting all downstream stages of infection progression

Upon HIV-1 infection of CD4^+^ T cells, NEAT1 expression is upregulated [68], leading to enhanced paraspeckle formation [66]. NEAT1 knockdown results in a decreased number of paraspeckles, while simultaneously enhancing the production of viral particles due to increased export of unspliced HIV-1 mRNAs from the nucleus to the cytoplasm [68] (Fig. 4A).

It is also important to note that NEAT1 expression levels are elevated in PBMCs from patients not receiving ART and decreased in patients receiving ART, with low NEAT1 levels correlating with CD4^+^ T cell counts [70]. This allows one to consider NEAT1 as a potential biomarker for monitoring an HIV infection [70].

**Long non-coding RNAs in viral evasion of the cellular immune response and DNA damage response**

**Fig. 5:**
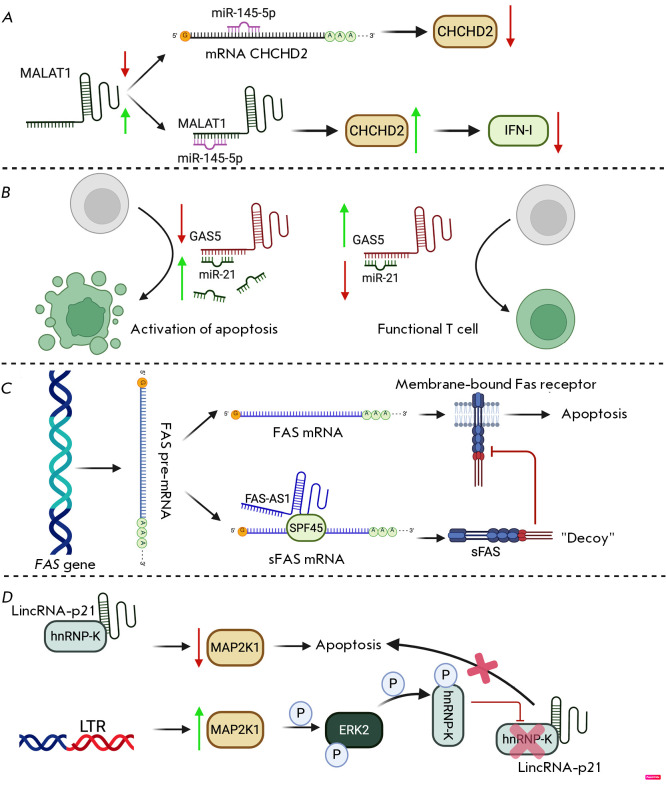
Schematic representation of the lncRNA-associated pathways of HIV-1 host immune evasion and apoptosis suppression. (A, B) GAS5 and MALAT1: regulation of the antiviral interferon response via the competing endogenous RNA (ceRNA) mechanism. (C) FAS-AS1: inhibition of apoptosis in infected macrophages via alternative splicing of the FAS receptor. (D) LincRNA-p21: suppression of the MAP2K1/ERK2 cell survival cascade via complex formation with the nuclear protein hnRNP-K and cascade activation in macrophages during the HIV-1 infection

It is impossible to pinpoint a single mechanism of action for the above lncRNAs. Collectively, they either interact with components of the host immune response or regulate the expression of pro- or anti-apoptotic genes (Fig. 5).

***GAS5.*** The lncRNA GAS5 (growth-arrest-specific transcript 5) plays an important role in the antiviral immune response and regulation of T-cell functions in the HIV infection. GAS5 expression is significantly decreased in HIV-1-infected cells [48]; however, it can still inhibit viral replication [76]. GAS5 can be classified as competing endogenous RNA (ceRNA). One of the key mechanisms of GAS5 antiviral action involves its ability to bind and suppress miR-873 activity. Overexpression of miR-873 results in a significant increase (>400-fold) in viral mRNA levels, while miR-873 inhibition using inhibitor miR-873-in reduces viral mRNA production by approximately 50%. Using the recombinant luciferase-encoding viral vector NL4-3, Chen L. et al. have shown that miR873 increases transcriptional activity from the HIV-1 LTR promoter approximately threefold. Therefore, miR-873 enhances transcription of viral genes, while GAS5, by binding to this miRNA, mimics the effect of miR-873 inhibition and ultimately inhibits HIV-1 replication [76].

Another mechanism by which GAS5 can modulate the HIV infection has also been described. GAS5 has been shown to downregulate miR-21 expression, thereby affecting the signaling pathways associated with DNA damage and programmed cell death [77]. An analysis of peripheral blood samples from HIV-1-patients receiving ART demonstrated reduced GAS5 expression and increased miR-21 levels in their CD4^+^ T cells. Downregulation of GAS5 and upregulation of miR-21 promote cell dysfunction and apoptosis (Fig. 5B). In patients receiving ART, persistent GAS5/miR-21 dysregulation contributes to chronic immune activation, as well as T-cell exhaustion and premature senescence, suggesting GAS5 as a potential therapeutic target for the HIV infection [77].

***MALAT1.*** The role of this lncRNA has already been discussed in the section on transcription modulation. However, studies using THP-1 monocyte-derived macrophages have shown that MALAT1 can also promote HIV-1 replication by acting as a ceRNA for CHCHD2 (coiled-coil-helix-coiled-coil-helix domain-containing 2) mRNA [78]. Expression of this gene is regulated by miR-145-5p, which binds to the 3’-untranslated region (3’-UTR) of CHCHD2 mRNA, thereby promoting its degradation via the RNA interference pathway [79]. MALAT1 contains a binding site for miR-145-5p. Therefore, by binding to miR-145-5p, MALAT1 rescues CHCHD2 mRNA from degradation and upregulates CHCHD2 expression in macrophages (Fig. 5A) [78]. CHCHD2 is a negative regulator of the innate immune response, since it suppresses the production of interferons α and β (IFNα/β), thereby reducing the effectiveness of the host antiviral response. Small-interfering-RNAmediated knockdown of both CHCHD2 and MALAT1 stimulates the expression of interferon regulatory factor 7 (IRF7), which activates IFNα/β gene expression, resulting in suppressed HIV-1 replication [78].

These findings improve our understanding of HIV-1 replication in macrophages via the MALAT1/miR145-5p/CHCHD2-mediated regulatory pathway and provide new therapeutic opportunities for the HIV-1 infection.

FAS-AS1. FAS-AS1, also known as SAF (FAS antisense 1), is a 1,536-nt-long lncRNA transcribed from the antisense strand of an intron within FAS at locus 10q23.31. FAS encodes the membrane-associated Fas receptor, also known as apoptosis antigen 1 (APO-1) and cluster of differentiation 95 (CD95), a member of the cell death receptor family. Its association with the membrane-bound glycoprotein FAS ligand (FasL) leads to receptor activation and cell apoptosis [80]. In contrast, FAS-AS1 prevents apoptosis by interacting with the splicing factor SPF45 and promoting alternative splicing of FAS mRNA. This yields a soluble form of the Fas receptor (sFAS), which binds FasL, thereby preventing FAS receptor activation and protecting cells from apoptosis (Fig. 5C) [81].

FAS-AS1 expression is significantly increased in HIV-1-infected human macrophages [82]. FAS-AS1 knockdown using siRNA disrupts alternative splicing of FAS and promotes Fas synthesis, which binds FasL, trimerizes, and forms the death-inducing signaling complex (DISC), thereby initiating apoptosis [82]. These properties of FAS-AS1 suggest its potential as a therapeutic target for the elimination of HIV-1- infected macrophages, which may reduce the number of viral reservoir cells.

***LincRNA-p21.*** LincRNA-p21 is a long intergenic non-coding RNA encoded at the human 6p21.2 locus in close proximity to the tumor suppressor gene p21, also known as CDKN1A (cyclin-dependent kinase inhibitor 1A) [37]. The term “intergenic” signifies that the corresponding gene does not overlap with any protein-coding gene. LincRNA-p21 has been identified as a transcriptional repressor regulated by the tumor suppressor p53, since inhibition of lincRNA-p21 expression stimulates transcription of genes that are normally repressed by p53 [83]. Indeed, lincRNA-p21 expression is regulated by p53, which binds to the lincRNA-p21 gene promoter and activates its transcription [83]. It has been shown that lincRNA-p21 regulates apoptosis, cell cycle progression at checkpoints, cell proliferation, reprogramming efficiency, and oncogenesis by modulating transcription, translation, chromatin remodeling, and energy metabolism [84].

One of the most important stages of the HIV-1 life cycle is the integration of viral cDNA into the infected cell’s genome. This process leads to DNA damage in the host genome, the repair of which involves the double-strand break (DSB) sensors ATM (ataxiatelangiectasia mutated) kinase and DNA-dependent protein kinase (DNA-PK) [85, 86]. If repair of the inflicted damage fails to occur, p53-mediated apoptosis is activated. HIV-1 induces apoptosis in CD4^+^ T cells but not in macrophages, which are major cellular reservoirs of the latent virus. This is due to the HIV-1- mediated targeted suppression of macrophage apoptosis involving lincRNA-p21 [87, 88].

In the absence of a viral infection, lincRNA-p21 forms a complex with the nuclear protein hnRNP-K (heterogeneous nuclear ribonucleoprotein K) in the cell nucleus. This complex functions as a transcriptional repressor of a wide range of pro-survival genes, including MAP2K1 [83, 89]. MAP2K1 (mitogen-activated protein kinase kinase 1) is the main kinase that activates ERK2 (extracellular signal-regulated kinase 2) in the canonical cell survival pathway [90]. Once activated, ERK2 phosphorylates hnRNP-K, which is subsequently translocated to the cytoplasm [88]. As a result, the nuclear lincRNA-p21–hnRNP-K complex fails to form, which stops the repression of survival genes (Fig. 5D).

In HIV-infected macrophages, MAP2K1 is upregulated, leading to ERK2 activation, hnRNP-K phosphorylation, and its inability to form a complex with lincRNA-p21. This ensures robust transcription of anti-apoptotic genes. This mechanism is supported by the fact that treatment of HIV-infected macrophages with MAP2K1 or ERK2 inhibitors induces apoptosis [87]. The pro-survival MAP2K1/ERK2 pathway is deactivated during T-cell maturation. Hence, infection of CD4^+^ T cells may lead to apoptosis. This indicates the possibility of reversing virus-mediated suppression of apoptosis by restoring the nuclear lincRNA-p21– hnRNP-K complex and suggests MAP2K1/ERK2 inhibitors as a potential therapeutic strategy for HIV-1 infection in macrophages.

Furthermore, HIV-1 utilizes the host RNA degradation apparatus to reduce lincRNA-p21 levels [87]. The RNA-binding protein HuR (human antigen R or ELAV-like protein 1) stabilizes mRNA by binding to AU-rich elements within the 3’-UTR and protecting it from degradation [91]. However, when HuR binds nuclear lincRNA-p21, it recruits the microRNA let-7 and protein Argonaute 2 (Ago2), a key RISC (RNAinduced silencing complex) component. This complex promotes lincRNA-p21 degradation via RNA interference [88, 92]. Under conditions of activated apoptosis, e.g., following DNA damage, HuR translocates to the cytoplasm and the nuclear level of lincRNA-p21 increases, stimulating the expression of pro-apoptotic genes. However, in HIV-infected macrophages, HuR remains in the nucleus, despite the presence of DNA damage.

The ability of HIV-1 to manipulate host lncRNA expression and function, particularly that of lincRNA-p21, represents a complex mechanism of immune evasion. Activation of the MAP2K1/ERK2 signaling pathway, together with nuclear localization of HuR, which induces lincRNA-p21 degradation, enables the virus to effectively counteract apoptosis in macrophages.

***NEAT1 and ZBTB11-AS1.*** In conclusion, we find it necessary to mention two other lncRNAs, whose involvement in the cellular immune response has been suggested but has yet to be confirmed. These lncRNAs are NEAT1, which has already been discussed, and ZBTB11-AS1 (zinc finger and BTB domain containing protein 11 antisense RNA 1), an antisense lncRNA complementary to ZBTB11 mRNA, which encodes a transcription factor associated with oncogenesis [93].

Using immortalized human microglial C20 cells, it has been shown that the expression levels and subcellular localization of NEAT1 and ZBTB11-AS1 vary depending on the stage of the HIV-1 infection [94]. During the active replication phase, when the viral genomic RNA and p24 protein levels reach their peak (day 4 post-infection), the total levels of these lncRNAs decrease. In contrast, during persistent infection (day 21 post-infection), which is characterized by minimal, yet detectable, cellular levels of viral RNA and p24 protein in the absence of viral RNA in the supernatant, the total levels of these lncRNAs increase. Both lncRNAs are detected in the nucleus and cytoplasm of microglial cells. However, on day 4 post-infection, NEAT1 levels increase in the cytoplasm and decrease in the nucleus. On day 21, cytoplasmic NEAT1 levels continue to increase, while nuclear NEAT1 levels remain decreased, and ZBTB11-AS1 demonstrates a decrease only in its nuclear level on day 4.

A bioinformatics analysis (using the NPInter and ShinyGO databases) predicted a potential association between NEAT1 and the proteins DDX3X (RNA helicase involved in HIV-1 RNA export and translation [95]) and ZC3HAV1 (a protein that promotes degradation of HIV-1 RNA [94, 96]). These proteins regulate the production of IL-6, a multifunctional cytokine involved in the immune and inflammatory responses [97]. An increase in IL-6 levels was found in the supernatant of microglial cells on day 4 post-infection, which may be due to NEAT1 translocation to the cytoplasm [94], where it can promote activation of the NLRP3 inflammasome and subsequent production of IL-6 [98, 99]. These data suggest that NEAT1 may be involved in the cellular immune response to a viral infection.

The bioinformatics analysis also predicted a possible interaction between ZBTB11-AS1 and the proteins DDX3X, ZC3HAV1, MOV10, and RBM15 [94]. MOV10 is an RNA helicase that binds to the RISC component Argonaute and plays an important role in the cellular response to a viral infection and suppression of retrotransposition [100]. RBM15 acts as a key regulator of N6-methyladenosine formation in RNA and participates in various cellular processes, including RNA splicing, nuclear export, and oncogenesis [101, 102]. These data suggest a possible role for ZBTB11-AS1 in modulating HIV-1 replication and the associated immune response through its interaction with multiprotein complexes regulating the stability, processing, and transport of viral transcripts, thus highlighting the importance of further studies into the role of this lncRNA in the HIV infection.

## CONCLUSIONS

The conducted analysis of the involvement of lncRNAs in the HIV-1 infection highlights their central role in the complex network of virus–host interactions and paints a multifaceted picture that determines the infection outcome. Long non-coding RNAs are more than just passive byproducts of transcription; they act as key regulators capable of exerting both proviral and antiviral effects. On the one hand, lncRNAs such as MALAT1, HEAL, uc002yug.2, and CYTOR serve as replication enhancers, promoting viral reactivation through disruption of the epigenetic landscape, recruitment of transcriptional activators (e.g., p300 and P-TEFb), modulation of splicing, and attenuation of the interferon response. On the other hand, lncRNAs NKILA, NRON, 7SK, and lincRNA-p21 act as suppressors of viral transcription, maintaining latency by either inhibiting key cellular factors (NF-κB, NFAT, and P-TEFb) or promoting apoptosis of infected cells. HIV-1 has evolved intricate strategies to thwart the antiviral functions of host lncRNAs, which emphasizes the dynamic nature of the virus–host interplay.

Thus, lncRNAs represent a fundamentally new class of potent therapeutic targets. Targeting specific lncRNAs may constitute a promising approach in the development of strategies for complete HIV-1 eradication. However, further in-depth studies are required to evaluate the specificity, efficacy, and safety of targeting lncRNAs in the heterogeneous cellular reservoirs of HIV-1.

## Abbreviations

ART: antiretroviral therapy
lncRNA: long non-coding RNA
LTR: long terminal repeat
LRA: latency-reversing agent
miRNA: microRNA
NF-κB: nuclear factor kappa-light-chain-enhancer of activated B cells
P-TEFb: positive transcription elongation factor b
PRC2: polycomb repressive complex 2
Tat: HIV trans-activator of transcription
TAR: trans-activation response element
ceRNA: competing endogenous RNA
SAHA: suberoylanilide hydroxamic acid
PBMCs: peripheral blood mononuclear cells
EZH2: enhancer of zeste homolog 2
SAMHD1: sterile alpha motif (SAM) and histidine-aspartic (HD) domain-containing protein 1
MAP2K1: mitogen-activated protein kinase kinase 1
siRNA: small interfering RNA
3’-UTR: 3’-untranslated region
